# Measles Contributes to Rheumatoid Arthritis: Evidence from Pathway and Network Analyses of Genome-Wide Association Studies

**DOI:** 10.1371/journal.pone.0075951

**Published:** 2013-10-18

**Authors:** Guiyou Liu, Yongshuai Jiang, Xiaoguang Chen, Ruijie Zhang, Guoda Ma, Rennan Feng, Liangcai Zhang, Mingzhi Liao, Yingbo Miao, Zugen Chen, Rong Zeng, Keshen Li

**Affiliations:** 1 Institute of Neurology, Guangdong Medical College, Zhanjiang, China; 2 Genome Analysis Laboratory, Tianjin Institute of Industrial Biotechnology, Chinese Academy of Sciences, Tianjin, China; 3 College of Bioinformatics Science and Technology, Harbin Medical University, Harbin, China; 4 Key Laboratory of Sweetpotato Biology and Genetic Breeding, Sweetpotato Research Institute, Chinese Academy of Agricultural Sciences, Xuzhou, Jiangsu, China; 5 Department of Nutrition and Food Hygiene, School of Public Health, Harbin Medical University, Harbin, China; 6 Department of Bioinformatics and Computational Biology, Division of Quantitative Sciences, The University of Texas M.D. Anderson Cancer Center, Houston, Texas, United States of America; 7 Department of Human Genetics, University of California Los Angeles, Los Angeles, California, United States of America; 8 Department of Orthopedic Surgery, Affiliated Hospital of Guangdong Medical College, Zhanjiang, China; Harvard Medical School, United States of America

## Abstract

Growing evidence from epidemiological studies indicates the association between rheumatoid arthritis (RA) and measles. However, the exact mechanism for this association is still unclear now. We consider that the strong association between both diseases may be caused by shared genetic pathways. We performed a pathway analysis of large-scale RA genome-wide association studies (GWAS) dataset with 5,539 cases and 20,169 controls of European descent. Meanwhile, we evaluated our findings using previously identified RA loci, protein-protein interaction network and previous results from pathway analysis of RA and other autoimmune diseases GWAS. We confirmed four pathways including Cytokine-cytokine receptor interaction, Jak-STAT signaling, T cell receptor signaling and Cell adhesion molecules. Meanwhile, we highlighted for the first time the involvement of Measles and Intestinal immune network for IgA production pathways in RA. Our results may explain the strong association between RA and measles, which may be caused by the shared genetic pathway. We believe that our results will be helpful for future genetic studies in RA pathogenesis and may significantly assist in the development of therapeutic strategies.

## Introduction

Rheumatoid arthritis (RA) is a chronic autoimmune disease characterized by inflammatory polyarthritis [1]. RA affects approximately 1% of the adult population worldwide [2]. According to the National Institute of Arthritis and Musculoskeletal and Skin Diseases, about 1.3 million adults in the U.S. suffer from RA [3]. RA is a complex disease caused by a combination of genetic susceptibility and environmental factors [4]. The complex genetic architecture of RA makes genetic analysis difficult. Recently, much effort has been devoted to finding common RA variants; especially genome-wide association studies (GWAS) [4], [5], [6], [7], [8], [9]. However, these known genetic factors just explain 50–60% of the genetic variance for susceptibility to ACPA-positive and 30–50% susceptibility for ACPA-negative RA [10]. Considering the complex genetic architecture, it is apparent that additional risk variants remain to be discovered.

Previous studies reported an increased antibody level to measles virus in RA patients [11]. The following studies confirmed the association between measles virus and RA. Rosenau BJ et al investigated 50 patients with rheumatoid factor (RF)-negative RA. The result showed that 11 of 50 (22%) samples had IgM antibodies to measles virus recombinant nucleoprotein [12]. Recently, Heijstek MW et al examined the persistence of measles antibodies between 400 juvenile idiopathic arthritis (JIA) patients and 2176 healthy controls aged 1–19 years. The results indicated that measles-specific geometric mean antibody concentrations in JIA patients were higher (*P*<0.001) compared with healthy controls [13].

**Figure 1:**
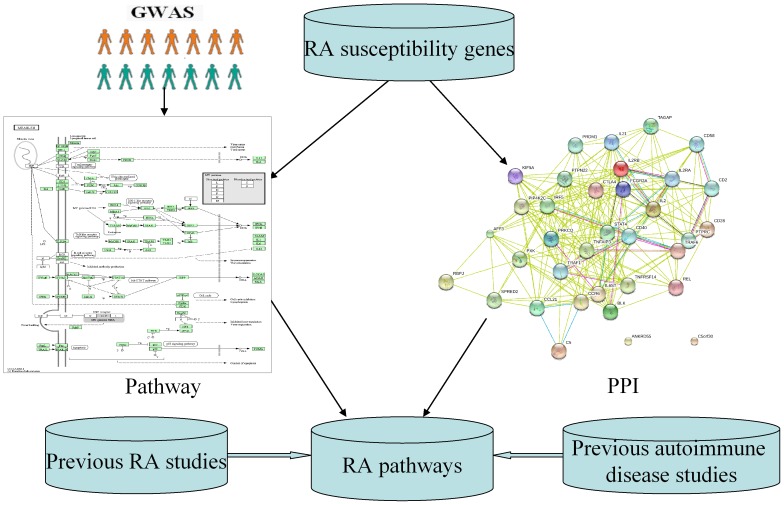
Flow chart of pathway and network analyses of RA GWAS.

Thus growing evidence from epidemiological studies indicates the association between RA and measles. However, the exact mechanism for this association is still unclear now. Both RA and measles are complex human diseases. We consider that the strong association between both diseases may be caused by shared genetic pathways. Fortunately, pathway-based method to study existing GWAS datasets has been applied into RA [3], [14], [15], [16], [17], [18], [19], [20]. However, no study reported the involvement of measles in RA. We found that all the pathway analyses used the Wellcome Trust Case-Control Consortium (WTCCC) or North American Rheumatoid Arthritis Consortium (NARAC) dataset or both datasets [3], [14], [15], [16], [17], [18], [19], [20]. We think that the study power would benefit from examining large-scale GWAS datasets. In this research, we performed a pathway analysis of large-scale RA GWAS dataset with 5,539 cases and 20,169 controls of European descent. Meanwhile, we evaluated our findings by combining existing knowledge including previously identified RA loci, protein-protein interaction network and previous results from pathway analysis of RA and other autoimmune diseases GWAS (Figure 1).

## Materials and Methods

### The RA GWAS dataset

The RA GWAS came from the meta-analysis of RA including 5,539 cases and 20,169 controls of European ancestry [8]. This study comprises six GWAS case-control collections, which included Brigham Rheumatoid Arthritis Sequential Study (BRASS, 483 cases and 1,449 controls), CANADA (589 cased and 1,472 controls), Epidemiological Investigation of Rheumatoid Arthritis (EIRA, 1,173 cased and 1,089 controls), NARAC I (867 cases and 1,041 controls), NARAC III (902 cased and 4,510 controls) and WTCCC (1,525 cased and 10,608 controls). Here, we used the summary results of this study for our analysis. A more detailed description can be obtained from the original publication [8].

### RA susceptibility genes identified by previous GWAS

We got 35 SNPs, which were validated or suggestive European RA risk associations, RA risk associations previously validated in East Asian case-control samples, and SNPs known to be highly differentiated across European populations [5], [21], [22], [23], [24], [25], [26], [27], [28]. Most SNPs have achieved *P*<5.00E-08 in combined analysis from previous studies [8]. The other SNPs have been validated by replication in independent samples but may not have attained *P*<5.00E-08 in any single study [5], [21], [22], [23], [24], [25], [26], [27], [28]. These 35 SNPs correspond to 35 RA susceptibility genes. More detailed information is described in Table S1 from the original study [8]. Considering that these findings were reported before 2010, we further accessed the GWAS Catalog (July 20, 2013) and selected 48 SNPs with *P*<5.00E-08, which corresponded to 41 susceptibility genes. In the end, we finally selected 57 RA susceptibility genes without duplication.

### Gene-based test for RA GWAS by VEGAS

A new gene-based test for GWAS approach, called VEGAS, was used to conduct a gene-based association study [29]. The method incorporates information from all SNPs within a gene and accounts for the gene sizes, SNP density, and the LD between SNPs. In brief, this method first assigns SNPs to each of 17,787 autosomal genes according to the positions on the UCSC Genome Browser hg18 assembly. SNPs within genes (including ±50 kb from the 5′ and 3′ UTR) are selected. Next, for a given gene with SNPs, association *P*-values are converted to uppertail chi-squared statistics with one degree of freedom (df). The gene-based test statistic is then the sum of all the chi-squared 1 df statistics within that gene. Then, multivariate normal simulations are used to account for the LD structure of SNPs within genes using the HapMap2 (CEU) genotype data [29]. In the first stage, 1000 simulations are performed. If the resulting empirical *P* value is less than 0.1, 10000 simulations are performed. If the empirical *P* value from 10000 simulations is less than 0.0001, the program will perform 1000000 simulations. For computational reasons, if the empirical *P* value is 0, then no more simulations will be performed. An empirical *P* value of 0 from 1000000 simulations can be interpreted as *P*<10E-06, which exceeds a Bonferroni-corrected threshold of *P*<2.8E-06 [∼0.05/17,787 (number of autosomal genes)] [30].

### Identifying RA risk pathways by hypergeometric test

We investigated the enrichment of RA genes identified by VEGAS and RA susceptibility genes identified by previous GWAS. For a given pathway in KEGG, hypergeometric test in Genecodis was used to detect an overrepresentation of the disease related genes among all the genes in the pathway [31]. The *P*-value of observing *K* disease related genes in the pathway can be calculated by
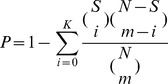
where *N* is the total number of genes that are of interest, *S* is the number of all disease related genes and *m* is the number of genes in the pathway, *K* is the number of disease related genes in the pathway.

GeneCodis (version 3) is a valuable tool to functionally interpret results from experimental techniques in genomics [32]. This web tool integrates different sources of information to finding groups of genes with similar biological meaning [32]. Effort has been made to remove noisy and redundant output from the enrichment results with the inclusion of a recently reported algorithm that summarizes significantly enriched terms and generates functionally coherent modules of genes and terms. A new comparative analysis has been added to allow the differential analysis of gene sets. New sources of biological information have been included, such as genetic diseases, drugs-genes interactions and Pubmed information among others [32].

### Comparison with previous pathway analyses of RA and other autoimmune disease

We compared our original findings with that of previous pathway analyses of RA GWAS [3], [14], [15], [16], [17], [18], [19], [20]. All studies used the WTCCC or NARAC dataset or both datasets. All the pathway analysis results are publicly available from the original studies or the corresponding supplementary materials [3], [14], [15], [16], [17], [18], [19], [20]. We also compared our original findings with that of previous pathway analyses of other autoimmune diseases including Crohn's disease (CD), celiac disease (CeID), type 1 diabetes (TID) and multiple sclerosis (MS). All the pathway analysis results are publicly available from the original studies or the corresponding supplementary materials [15], [20], [33], [34], [35], [36].

### Protein-protein interaction network analysis by STRING

We investigated the potential interactions between the proteins encoded by RA susceptibility genes using Search Tool for the Retrieval of Interacting Genes (STRING) (version 9.0). STRING is a database of known and predicted protein interactions, including direct (physical) and indirect (functional) associations, which are derived from four sources including genomic context, high-throughput experiments, coexpression and previous knowledge [37]. STRING provides uniquely comprehensive coverage and ease of access to both experimental as well as predicted interaction information. Interactions in STRING are provided with a confidence score, and accessory information such as protein domains and 3D structures is made available, all within a stable and consistent identifier space. New features in STRING include an interactive network viewer that can cluster networks on demand, updated on-screen previews of structural information including homology models, extensive data updates and strongly improved connectivity and integration with third-party resources. Version 9.0 of STRING covers more than 1100 completely sequenced organisms [37]. Recent publication describes the update to version 9.1 of STRING, introducing several improvements including the automated mining of scientific texts for interaction information, to now also include full-text articles, re-designed the algorithm for transferring interactions from one model organism to the other; and statistical information on any functional enrichment observed in networks [38].

## Results

### Pathway analysis of RA GWAS

After removal of the Major histocompatibility complex (MHC) region, we got 394 RA genes with adjusted *P*<0.01 by VEGAS. We then conducted a KEGG pathway analysis of these 394 significant RA genes. 152 KEGG pathways, which included at least one of 394 RA genes, were available for analysis. We identified 12 significant pathways with *P*< = 1.00E-03 after False discovery rate (FDR) multiple testing corrections (The threshold indicating significant results is 0.05), among which Measles (hsa05162) was the most significant signal (*P* = 1.57E-08) (Table 1). All detailed results are described in Table S1.

**Table 1:** Significant pathways with *P*< = 0.001 by pathway analysis of RA GWAS.

Pathway ID	Pathway Name	Significant genes	Gene in pathway	*P*	FDR
hsa05162	Measles	14	130	1.20E-10	1.57E-08
hsa04660	T cell receptor signaling pathway	11	107	1.95E-08	1.28E-06
hsa04060	Cytokine-cytokine receptor interaction	15	259	1.32E-07	5.75E-06
hsa05223	Non-small cell lung cancer	7	53	1.40E-06	4.57E-05
hsa05152	Tuberculosis	11	172	2.41E-06	6.32E-05
hsa04630	Jak-STAT signaling pathway	10	153	5.71E-06	1.25E-04
hsa04672	Intestinal immune network for IgA production	6	44	6.67E-06	1.25E-04
hsa04514	Cell adhesion molecules (CAMs)	9	125	7.67E-06	1.26E-04
hsa05212	Pancreatic cancer	7	70	9.32E-06	1.36E-04
hsa05200	Pathways in cancer	14	324	1.06E-05	1.38E-04
hsa04640	Hematopoietic cell lineage	7	83	2.87E-05	3.42E-04
hsa04650	Natural killer cell mediated cytotoxicity	8	125	5.73E-05	6.26E-04

### Pathway of RA susceptibility genes

We conducted a pathway analysis of 35 RA susceptibility genes identified by previous GWAS in European descent. 45 KEGG pathways, which included at least one of 35 RA genes, were available for analysis. After FDR multiple testing corrections (The threshold indicating significant results is 0.05), we identified 10 significant pathways (*P*< = 0.001), which contained at least three RA susceptibility genes. Interestingly, the results supported our previous findings. 6 pathways identified by pathway-based test of RA GWAS were replicated. These pathways included T cell receptor signaling (hsa04660), Cytokine-cytokine receptor interaction (hsa04060), Measles (hsa05162), Jak-STAT signaling (hsa04630), Cell adhesion molecules (hsa04514) and Intestinal immune network for IgA production (hsa04672), among which Cytokine-cytokine receptor interaction was the most significant pathway (*P* = 1.90E-10) and Measles was the second significant pathway (*P* = 1.35E-09) (Table 2).

**Table 2:** Significant pathways with *P*< = 0.001 by pathway analysis of RA susceptibility genes.

Pathway ID	Pathway Name	Significant genes	Gene in pathway	*P*	FDR
hsa04060	Cytokine-cytokine receptor interaction	9	259	4.23E-12	1.90E-10
hsa05162	Measles	7	130	5.98E-11	1.35E-09
hsa04630	Jak-STAT signaling pathway	7	153	1.89E-10	2.83E-09
hsa04514	Cell adhesion molecules	6	125	3.14E-09	3.53E-08
hsa04660	T cell receptor signaling pathway	5	107	8.21E-08	7.39E-07
hsa05320	Autoimmune thyroid disease	4	45	1.33E-07	9.96E-07
hsa05322	Systemic lupus erythematosus	4	88	2.01E-06	1.29E-05
hsa05330	Allograft rejection	3	29	3.52E-06	1.98E-05
hsa04672	Intestinal immune network for IgA production	3	44	1.26E-05	6.31E-05
hsa04620	Toll-like receptor signaling pathway	3	101	1.53E-04	6.87E-04

### Comparison with previous pathway analyses of RA and other autoimmune diseases

After comparison with previous pathway analyses of RA GWAS, we confirmed our original findings. Four pathways including Cytokine-cytokine receptor interaction (hsa04060), Jak-STAT signaling pathway (hsa04630), T cell receptor signaling (hsa04660) and Cell adhesion molecules (hsa04514) were identified by at least one pathway analysis. Among the pathway analysis methods, cumulative trend test adjusts for gene length by permutations and LD based gene block method [15], [18]. Here, we list the pathways, methods, the corresponding pathway *P*-values and references (Table 3). For more detailed information, please refer to the original studies [3], [14], [15], [16], [17], [18], [19], [20].

**Table 3:** Summary of the available results for pathway analyses of RA GWAS.

Pathway	Dataset	Method	*P*-value	Ref
Cytokine-cytokine receptor interaction	WTCCC	Decorrelation test (Fisher)	<1.00E-17	[16]
Cytokine-cytokine receptor interaction	NARAC	Decorrelation test (Fisher)	<1.00E-17	[16]
Cytokine-cytokine receptor interaction	NARAC WTCCC	Prioritizer (Bayesian approach)	4.33E-02	[17]
Cytokine-cytokine receptor interaction	WTCCC	Cumulative trend test	1.00E-42	[18]
Cytokine-cytokine receptor interaction	NARAC	Cumulative trend test	1.00E-29	[18]
Cytokine-cytokine receptor interaction	WTCCC	Cumulative trend test	2.00E-03	[15]
Jak-STAT signaling pathway	WTCCC	Decorrelation test (Fisher)	1.55E-10	[16]
Jak-STAT signaling pathway	NARAC	Decorrelation test (Fisher)	<1.00E-17	[16]
Jak-STAT signaling pathway	NARAC WTCCC	Prioritizer (Bayesian approach)	6.70E-03	[17]
Jak-STAT signaling pathway	WTCCC	Cumulative trend test	3.89E-15	[18]
Jak-STAT signaling pathway	NARAC	Cumulative trend test	1.30E-12	[18]
Jak-STAT signaling pathway	WTCCC	Cumulative trend test	4.40E-09	[15]
Jak-STAT signaling pathway	WTCCC	ClueGO (hypergeometric test)	7.41E-03	[19]
T cell receptor signaling pathway	WTCCC	Cumulative trend test	1.00E-211	[18]
T cell receptor signaling pathway	NARAC	Cumulative trend test	1.00E-331	[18]
T cell receptor signaling pathway	NARAC WTCCC	Prioritizer (Bayesian approach)	2.33E-02	[17]
T cell receptor signaling pathway	WTCCC	ClueGO (hypergeometric test)	2.70E-05	[19]
Cell adhesion molecules	NARAC	Binomial test	2.40E-04	[3]
Cell adhesion molecules	NARAC	Random set	<1.00E-04	[3]
Cell adhesion molecules	NARAC	Chi-square test	2.00E-02	[14]
Cell adhesion molecules	NARAC	DirEV test	5.00E-03	[14]
Cell adhesion molecules	NARAC	IndirEV test	4.00E-03	[14]
Cell adhesion molecules	WTCCC	Cumulative trend test	<1.00E-04	[15]
Cell adhesion molecules	WTCCC	Linear combination test	2.77E-11	[16]
Cell adhesion molecules	NARAC	Linear combination test	<1.00E-17	[16]
Cell adhesion molecules	WTCCC	Quadratic test	<1.00E-17	[16]
Cell adhesion molecules	NARAC	Quadratic test	<1.00E-17	[16]
Cell adhesion molecules	WTCCC	Decorrelation test (FDR)	3.63E-17	[16]
Cell adhesion molecules	NARAC	Decorrelation test (FDR)	8.65E-17	[16]
Cell adhesion molecules	WTCCC	Decorrelation test (Fisher)	<1.00E-17	[16]
Cell adhesion molecules	NARAC	Decorrelation test (Fisher)	<1.00E-17	[16]
Cell adhesion molecules	NARAC WTCCC	Prioritizer (Bayesian approach)	2.33E-02	[17]
Cell adhesion molecules	WTCCC	Cumulative trend test	1.00E-68	[18]
Cell adhesion molecules	NARAC	Cumulative trend test	1.00E-289	[18]
Cell adhesion molecules	WTCCC	BINGO (hypergeometric test)	6.06E-06	[20]

Abbreviations: RA, rheumatoid arthritis; WTCCC, Wellcome Trust Case-Control Consortium; NARAC, North American Rheumatoid Arthritis Consortium.

In addition to the involvement of these four pathways in RA, the associations between the four pathways and other autoimmune diseases also have been reported by previous pathway analyses, such as Cytokine-cytokine receptor interaction (hsa04060) in Crohn's disease (CD) [15], [33] and celiac disease (CeID) [36], Jak-STAT signaling pathway (hsa04630) in CD [15], [33], [34], T cell receptor signaling (hsa04660) in CD [34], Cell adhesion molecules (hsa04514) in CD [15], type 1 diabetes (TID) [15], [20] and multiple sclerosis (MS) [20], [35].

### Network analysis of RA susceptibility genes and RA GWAS

**Figure 2:**
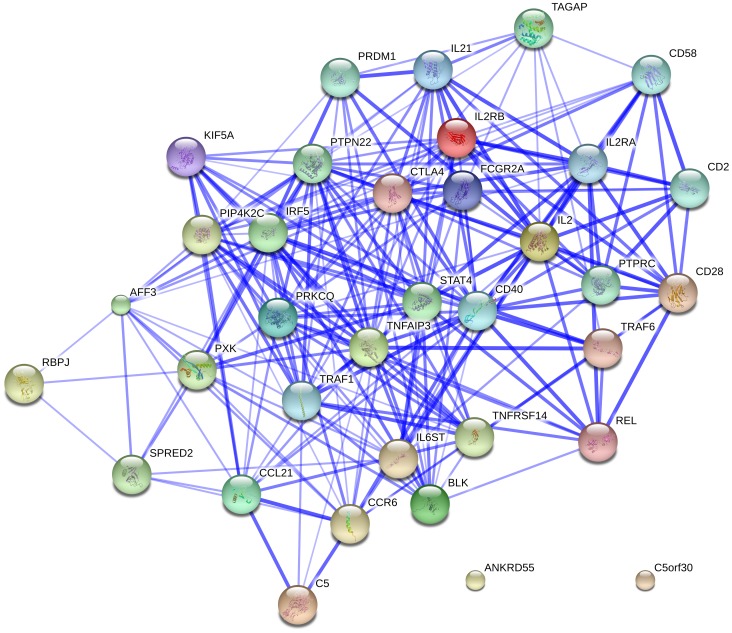
Network of known and predicted interactions between proteins encoded by 57 RA susceptibility genes.

**Figure 3:**
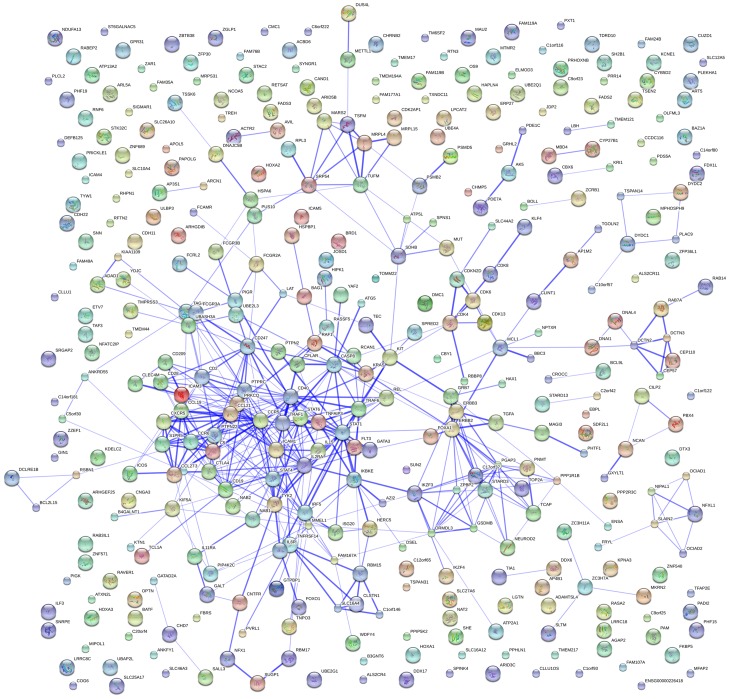
Network of known and predicted interactions between proteins encoded by 394 RA susceptibility genes identified by GWAS.

Based on the results above, we confirmed four RA risk pathways and highlighted for the first time the involvement of Measles and Intestinal immune network for IgA production pathways in RA. In order to further verify these findings, we conducted a protein-protein interaction network analysis of 35 RA susceptibility genes. Interestingly, we found significant connectivity among proteins encoded by RA susceptibility genes according to protein-protein interaction network in STRING (9.0) using default settings (observed interaction, 103; expected interaction, 1.88; *P*<1.00E-10) (Figure 2). Using the 394 RA susceptibility genes from RA GWAS, we further found significant connectivity among proteins encoded by these genes (observed interaction, 471; expected interaction, 4.36; *P*<1.00E-10) (Figure 3). Meanwhile, the results showed interaction between the Measles and Intestinal immune network for IgA production pathways and another four RA risk pathways.

### Verification by PubMed and Google Scholar literature search

In order to verify our findings, we searched the PubMed and Google Scholar databases. The results indicated that genes involved in the measles pathway were associated with RA. The evidence from literature search was described in Table 4.

**Table 4:** Literature evidences supporting that genes in measles pathway are associated with RA.

Gene	Supporting evidence	Ref
TNFAIP3	In conclusion, we have demonstrated an increase in TNFAIP3 expression in PBMCs from patients with RA compared with healthy controls	[57]
TNFAIP3	Together, these observations indicate a critical and cell-specific function for TNFAIP3 (A20) in the etiology of rheumatoid arthritis, supporting the idea of developing A20 modulatory drugs as cell-targeted therapies.	[58]
IL2	Our results replicate and firmly establish the association of STAT4 and CTLA4 with RA and provide highly suggestive evidence for IL2/IL21 loci as a risk factor for RA.	[59]
IL2	The KIAA1109-TENR-IL2-IL21 gene cluster, that encodes an interleukin (IL-21) that plays an important role in Th17 cell biology, is the 20th locus for which there is a genome-wide (P<or = 5×10(−8)) level of support for association with RA.	[60]
IL2RA	The present genetic and serologic data suggest that inherited altered genetic constitution at the IL2RA locus may predispose to a less destructive course of RA.	[61]
CD28	Modulation of CD28 expression with anti-tumor necrosis factor alpha therapy in rheumatoid arthritis	[62]
*STAT1*	Activation of the STAT1 pathway in rheumatoid arthritis	[63]
Tyk2	Our results demonstrate a critical contribution of Tyk2 in the development of arthritis, and we propose that Tyk2 might be an important candidate for drug development.	[64]
IKBKE	Combination therapy with low dose IFNβ and an IKBKE inhibitor might improve efficacy of either agent alone and offers a novel approach to RA.	[65]

Recently, Heijstek et al. investigated the effects of live attenuated measles-mumps-rubella (MMR) vaccination on disease activity in juvenile idiopathic arthritis (JIA) patients [39]. 137 patients with JIA aged 4 to 9 years were available for analysis. Patients were randomly assigned to receive MMR booster vaccination (n = 68) or no vaccination (control group; n = 69). At 12 months, seroprotection rates were higher in revaccinated patients vs. controls, as were antibody concentrations against measles, mumps and rubella [39].

## Discussion

Here, considering the association between RA and measles reported by previous epidemiological studies, we investigated the shared genetic pathways by integrating GWAS with pathway and protein-protein interaction network. In the end, we confirmed four RA risk pathways reported by previous pathway analyses, including Cytokine-cytokine receptor interaction pathway, Jak-STAT signaling pathway, T cell receptor signaling pathway and Cell adhesion molecules pathway. Until now, targeting two of these four pathways is also a reality. For the Cytokine-cytokine receptor interaction pathway, a broad range of cytokines are active in the joints of RA patients [40]. Anti-cytokine therapy of RA have been proposed, among which anti-TNF therapy of RA has proved effective [41]. Meanwhile, anti-other cytokines can also be beneficial, such as many interleukin (IL) family genes including IL-1, IL-6, IL-23, and IL-2 families [40].

For the Jak-STAT signaling pathway, many cytokines exert their effect by the Jak-STAT signal transduction [42]. Functional inhibition of JAK3 is sufficient for efficacy in collagen-induced arthritis in mice [43]. Targeting JAKs is also a reality in RA, such as tofacitinib targeting JAK1, JAK2 and JAK3 to treat RA (Phase III), VX-509 and R-348 targeting JAK3 to treat RA (Phase II) [44]. Recently, two studies examine the effects of JAK inhibitor tofacitinib in RA to elucidate the role of JAK in disease process [45], [46]. The results show that tofacitinib regulates RA synovitis through inhibition of interferon-γ and IL-17 production [45]. JAK inhibition with tofacitinib also suppresses arthritic joint structural damage through decreased receptor activator of NF-κB ligand (RANKL) production [46].

For the T cell receptor signaling pathway, evidence is emerging that altered T cell receptor signaling thresholds could contribute to human autoimmune arthritis, including RA and the spondyloarthropathies (SpA) [47]. T cell activation has been identified in RA pathogenesis by RA GWAS [48]. It is also reported that defective activation of T cell receptor-proximal signaling proteins, such as small GTPase Rap1, contribute to the pathologic behavior of RA synovial T cells [49]. Evidence indicated that maintenance of T cell Rap1 signaling in murine T cells could reduce disease incidence and severity in collagen-induced arthritis [49]. T cell receptor signal strength also controls arthritis severity in proteoglycan-specific TCR transgenic mice [50].

For the Cell adhesion molecules pathway, the elevated production of cell adhesion molecules is crucial to the pathogenesis of RA [51]. Some cell adhesion molecules, such as E-selectin and intercellular adhesion molecule-1 (ICAM-1) are modulated in RA patients who respond clinically to drug treatment [52]. Recently, Klimiuk PA et al. analyzed serum concentrations of soluble intercellular adhesion molecule-1 (sICAM-1), vascular cell adhesion molecule-1 (sVCAM-1), and E-selectin (sE-selectin) in patients with early rheumatoid arthritis (RA) before and after 6 months of treatment with methotrexate (MTX). The results indicated that patients with early RA were characterized by high serum concentrations of sICAM-1, sVCAM-1, and sE-selectin. Therapy with MTX resulted in clinical improvement and diminished serum levels of soluble cell adhesion molecules in the RA patients [53].

Growing evidence from epidemiological studies indicates the association between RA and measles. An increased antibody level to measles virus was observed in JIA patients vs. healthy controls [11], [13]. Recently, Heijstek et al. investigated the effects of live attenuated measles-mumps-rubella (MMR) vaccination on disease activity in JIA patients [39]. 137 patients with JIA aged 4 to 9 years were available for analysis. Patients were randomly assigned to receive MMR booster vaccination (n = 68) or no vaccination (control group; n = 69). At 12 months, seroprotection rates were higher in revaccinated patients vs. controls, as were antibody concentrations against measles, mumps and rubella [39].

We highlighted for the first time the involvement of Measles in RA by pathway analysis method. We further confirmed these findings by previously identified RA loci, protein-protein interaction network. Our results may explain the strong association between RA and measles, which may be caused by shared genetic pathway. We believe that our results will be helpful for future genetic studies in RA pathogenesis and may significantly assist in the development of therapeutic strategies by targeting the Measles pathway and reducing antibody level to measles virus in RA. Future replication studies using animal models are required to replicate our findings before the findings can be of clinical use.

Here, we analyzed 25,708 individuals from European population. The study power would benefit from examining this large-scale RA GWAS dataset, which was originally analyzed by Stahl EA et al. [8]. The power was calculated based on the odds ratios under a multiplicative genotypic relative risk (GRR) model at four thresholds for significant SNPs with 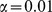, 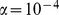, 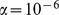 and 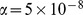. The results indicated that this study had a great power to detect genetic associations at 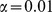. For heterozygote GRR of 1.1, significant SNPs with 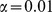 and minor allele frequency (MAF) = 0.2 had a power 0.8. For heterozygote GRR of 1.2, significant SNPs with 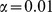 and minor allele frequency (MAF) = 0.1 had a power 1. More detailed information was described in the supplementary materials of the original study [8]. These results were consistent with recent findings. Recently, Hunt et al. genotyped 25 GWAS risk genes in 41,911 UK residents of white European origin (24,892 cases with six autoimmune diseases and 17,019 controls) [54]. The results showed that the missing heritability for common autoimmune diseases may be a result of many common-variant loci of weak effect [54]. Meanwhile, in order to reduce the sources of bias in pathway analysis, we adjusted gene length and LD patterns in human genome using VEGAS, which increased the reliability of our results. Our findings indicate that integration of GWAS dataset with pathway and protein-protein interaction network can uncover novel RA risk pathways. We think that this strategy may be applied into other phenotypes or diseases.

In our research, we selected hypergeometric test for pathway analysis, which is a commonly used competitive test that is incorporated in a number of bioinformatics tools as described in the review paper about the genome-wide pathway analysis to unravel the etiology of complex diseases [55]. In Table 3, pathway analysis software ClueGO and BINGO were also used hypergeometric test. Jia et al. suggested that for gene sets consisting of markers highly associated (*P*< = 1.00E-03) with disease, among gene set enrichment analysis (GSEA), hypergeometric test and SNP ratio test (SRT), the hypergeometric test performed best with the highest power [56].

Our study also has some limitations. First, except the human studies, there was no any animal study, which investigated the measles related genes and RA before. Second, we identified the involvement of Measles in RA by pathway analysis of RA GWAS. Until now, no evidence indicates that Measles pathway is significantly enriched for genetic association to Measles by pathway analysis of Measles GWAS. Considering these limitations, we will replicate this pathway by pathway analysis of Measles GWAS in future work, when Measles GWAS is available for us.

## Supporting Information

Table S1 **The detailed results from pathway analysis of RA GWAS.**
(DOC)

## References

[1] Raychaudhuri S, Thomson BP, Remmers EF, Eyre S, Hinks A, et al (2009) Genetic variants at CD28, PRDM1 and CD2/CD58 are associated with rheumatoid arthritis risk. Nat Genet 41: 1313–1318. 19898481 10.1038/ng.479 PMC3142887

[2] Cerhan JR, Saag KG, Merlino LA, Mikuls TR, Criswell LA (2003) Antioxidant micronutrients and risk of rheumatoid arthritis in a cohort of older women. Am J Epidemiol 157: 345–354. 12578805 10.1093/aje/kwf205

[3] Ballard DH, Aporntewan C, Lee JY, Lee JS, Wu Z, et al (2009) A pathway analysis applied to Genetic Analysis Workshop 16 genome-wide rheumatoid arthritis data. BMC Proc 3 Suppl 7: S91. 20018088 10.1186/1753-6561-3-s7-s91 PMC2795995

[4] Plenge RM, Seielstad M, Padyukov L, Lee AT, Remmers EF, et al (2007) TRAF1-C5 as a risk locus for rheumatoid arthritis–a genomewide study. N Engl J Med 357: 1199–1209. 17804836 10.1056/NEJMoa073491 PMC2636867

[5] The Wellcome Trust Case Control Consortium (2007) Genome-wide association study of 14,000 cases of seven common diseases and 3,000 shared controls. Nature 447: 661–678. 17554300 10.1038/nature05911 PMC2719288

[6] Gregersen PK, Amos CI, Lee AT, Lu Y, Remmers EF, et al (2009) REL, encoding a member of the NF-kappaB family of transcription factors, is a newly defined risk locus for rheumatoid arthritis. Nat Genet 41: 820–823. 19503088 10.1038/ng.395 PMC2705058

[7] Freudenberg J, Lee HS, Han BG, Shin HD, Kang YM, et al (2011) Genome-wide association study of rheumatoid arthritis in Koreans: population-specific loci as well as overlap with European susceptibility loci. Arthritis Rheum 63: 884–893. 21452313 10.1002/art.30235

[8] Stahl EA, Raychaudhuri S, Remmers EF, Xie G, Eyre S, et al (2010) Genome-wide association study meta-analysis identifies seven new rheumatoid arthritis risk loci. Nat Genet 42: 508–514. 20453842 10.1038/ng.582 PMC4243840

[9] Okada Y, Terao C, Ikari K, Kochi Y, Ohmura K, et al (2012) Meta-analysis identifies nine new loci associated with rheumatoid arthritis in the Japanese population. Nat Genet 44: 511–516. 22446963 10.1038/ng.2231

[10] de Vries R (2011) Genetics of rheumatoid arthritis: time for a change!. Curr Opin Rheumatol 23: 227–232. 21427575 10.1097/BOR.0b013e3283457524

[11] Kalliomaki JL, Halonen P, Salmi A (1975) Virus antibodies in serum and synovial fluid of patients with rheumatoid arthritis and other connective tissue diseases. Ann Rheum Dis 34: 43–48. 1124954 10.1136/ard.34.1.43 PMC1006343

[12] Rosenau BJ, Schur PH (2009) Association of measles virus with rheumatoid arthritis. J Rheumatol 36: 893–897. 19435971 10.3899/jrheum.080856

[13] Heijstek MW, van Gageldonk PG, Berbers GA, Wulffraat NM (2012) Differences in persistence of measles, mumps, rubella, diphtheria and tetanus antibodies between children with rheumatic disease and healthy controls: a retrospective cross-sectional study. Ann Rheum Dis 71: 948–954. 22172491 10.1136/annrheumdis-2011-200637

[14] Beyene J, Hu P, Hamid JS, Parkhomenko E, Paterson AD, et al (2009) Pathway-based analysis of a genome-wide case-control association study of rheumatoid arthritis. BMC Proc 3 Suppl 7: S128. 20017994 10.1186/1753-6561-3-s7-s128 PMC2795901

[15] Eleftherohorinou H, Wright V, Hoggart C, Hartikainen AL, Jarvelin MR, et al (2009) Pathway analysis of GWAS provides new insights into genetic susceptibility to 3 inflammatory diseases. PLoS One 4: e8068. 19956648 10.1371/journal.pone.0008068 PMC2778995

[16] Luo L, Peng G, Zhu Y, Dong H, Amos CI, et al (2010) Genome-wide gene and pathway analysis. Eur J Hum Genet 18: 1045–1053. 20442747 10.1038/ejhg.2010.62 PMC2924916

[17] Martin JE, Alizadeh BZ, Gonzalez-Gay MA, Balsa A, Pascual-Salcedo D, et al (2010) Identification of the oxidative stress-related gene MSRA as a rheumatoid arthritis susceptibility locus by genome-wide pathway analysis. Arthritis Rheum 62: 3183–3190. 20617525 10.1002/art.27648

[18] Eleftherohorinou H, Hoggart CJ, Wright VJ, Levin M, Coin LJ (2011) Pathway-driven gene stability selection of two rheumatoid arthritis GWAS identifies and validates new susceptibility genes in receptor mediated signalling pathways. Hum Mol Genet 20: 3494–3506. 21653640 10.1093/hmg/ddr248

[19] Bakir-Gungor B, Sezerman OU (2011) A new methodology to associate SNPs with human diseases according to their pathway related context. PLoS One 6: e26277. 22046267 10.1371/journal.pone.0026277 PMC3201947

[20] Baranzini SE, Galwey NW, Wang J, Khankhanian P, Lindberg R, et al (2009) Pathway and network-based analysis of genome-wide association studies in multiple sclerosis. Hum Mol Genet 18: 2078–2090. 19286671 10.1093/hmg/ddp120 PMC2678928

[21] Eyre S, Flynn E, Martin P, Hinks A, Wilson AG, et al (2010) No evidence for association of the KLF12 gene with rheumatoid arthritis in a large UK cohort. Annals of the Rheumatic Diseases 69: 1407–1408. 19907058 10.1136/ard.2009.120428

[22] Julia A, Ballina J, Canete JD, Balsa A, Tornero-Molina J, et al (2008) Genome-wide association study of rheumatoid arthritis in the Spanish population: KLF12 as a risk locus for rheumatoid arthritis susceptibility. Arthritis and Rheumatism 58: 2275–2286. 18668548 10.1002/art.23623

[23] Lee YC, Raychaudhuri S, Cui J, De Vivo I, Ding B, et al (2009) The PRL −1149 G/T polymorphism and rheumatoid arthritis susceptibility. Arthritis and Rheumatism 60: 1250–1254. 19404952 10.1002/art.24468 PMC2956274

[24] Raychaudhuri S, Remmers EF, Lee AT, Hackett R, Guiducci C, et al (2008) Common variants at CD40 and other loci confer risk of rheumatoid arthritis. Nature Genetics 40: 1216–1223. 18794853 10.1038/ng.233 PMC2757650

[25] Raychaudhuri S, Thomson BP, Remmers EF, Eyre S, Hinks A, et al (2009) Genetic variants at CD28, PRDM1 and CD2/CD58 are associated with rheumatoid arthritis risk. Nature Genetics 41: 1313–1318. 19898481 10.1038/ng.479 PMC3142887

[26] Kochi Y, Yamada R, Suzuki A, Harley JB, Shirasawa S, et al (2005) A functional variant in FCRL3, encoding Fc receptor-like 3, is associated with rheumatoid arthritis and several autoimmunities. Nature Genetics 37: 478–485. 15838509 10.1038/ng1540 PMC1362949

[27] Suzuki A, Yamada R, Chang X, Tokuhiro S, Sawada T, et al (2003) Functional haplotypes of PADI4, encoding citrullinating enzyme peptidylarginine deiminase 4, are associated with rheumatoid arthritis. Nature Genetics 34: 395–402. 12833157 10.1038/ng1206

[28] Suzuki A, Yamada R, Kochi Y, Sawada T, Okada Y, et al (2008) Functional SNPs in CD244 increase the risk of rheumatoid arthritis in a Japanese population. Nature Genetics 40: 1224–1229. 18794858 10.1038/ng.205

[29] Liu JZ, McRae AF, Nyholt DR, Medland SE, Wray NR, et al (2010) A versatile gene-based test for genome-wide association studies. Am J Hum Genet 87: 139–145. 20598278 10.1016/j.ajhg.2010.06.009 PMC2896770

[30] Cornelis MC, Monda KL, Yu K, Paynter N, Azzato EM, et al (2011) Genome-wide meta-analysis identifies regions on 7p21 (AHR) and 15q24 (CYP1A2) as determinants of habitual caffeine consumption. PLoS Genet 7: e1002033. 21490707 10.1371/journal.pgen.1002033 PMC3071630

[31] Nogales-Cadenas R, Carmona-Saez P, Vazquez M, Vicente C, Yang X, et al (2009) GeneCodis: interpreting gene lists through enrichment analysis and integration of diverse biological information. Nucleic Acids Res 37: W317–322. 19465387 10.1093/nar/gkp416 PMC2703901

[32] Tabas-Madrid D, Nogales-Cadenas R, Pascual-Montano A (2012) GeneCodis3: a non-redundant and modular enrichment analysis tool for functional genomics. Nucleic Acids Res 40: W478–483. 22573175 10.1093/nar/gks402 PMC3394297

[33] Gui H, Li M, Sham PC, Cherny SS (2011) Comparisons of seven algorithms for pathway analysis using the WTCCC Crohn's Disease dataset. BMC Res Notes 4: 386. 21981765 10.1186/1756-0500-4-386 PMC3199264

[34] Wang K, Zhang H, Kugathasan S, Annese V, Bradfield JP, et al (2009) Diverse genome-wide association studies associate the IL12/IL23 pathway with Crohn Disease. American Journal of Human Genetics 84: 399–405. 19249008 10.1016/j.ajhg.2009.01.026 PMC2668006

[35] Wang JH, Pappas D, De Jager PL, Pelletier D, de Bakker PI, et al (2011) Modeling the cumulative genetic risk for multiple sclerosis from genome-wide association data. Genome Med 3: 3. 21244703 10.1186/gm217 PMC3092088

[36] Trynka G, Wijmenga C, van Heel DA (2010) A genetic perspective on coeliac disease. Trends Mol Med 16: 537–550. 20947431 10.1016/j.molmed.2010.09.003

[37] Szklarczyk D, Franceschini A, Kuhn M, Simonovic M, Roth A, et al (2011) The STRING database in 2011: functional interaction networks of proteins, globally integrated and scored. Nucleic Acids Res 39: D561–568. 21045058 10.1093/nar/gkq973 PMC3013807

[38] Franceschini A, Szklarczyk D, Frankild S, Kuhn M, Simonovic M, et al (2013) STRING v9.1: protein-protein interaction networks, with increased coverage and integration. Nucleic Acids Res 41: D808–815. 23203871 10.1093/nar/gks1094 PMC3531103

[39] Heijstek MW, Kamphuis S, Armbrust W, Swart J, Gorter S, et al (2013) Effects of the live attenuated measles-mumps-rubella booster vaccination on disease activity in patients with juvenile idiopathic arthritis: a randomized trial. JAMA 309: 2449–2456. 23780457 10.1001/jama.2013.6768

[40] Brennan FM, McInnes IB (2008) Evidence that cytokines play a role in rheumatoid arthritis. J Clin Invest 118: 3537–3545. 18982160 10.1172/JCI36389 PMC2575731

[41] Gaestel M, Kotlyarov A, Kracht M (2009) Targeting innate immunity protein kinase signalling in inflammation. Nat Rev Drug Discov 8: 480–499. 19483709 10.1038/nrd2829

[42] Seidel HM, Lamb P, Rosen J (2000) Pharmaceutical intervention in the JAK/STAT signaling pathway. Oncogene 19: 2645–2656. 10851064 10.1038/sj.onc.1203550

[43] Lin TH, Hegen M, Quadros E, Nickerson-Nutter CL, Appell KC, et al (2010) Selective functional inhibition of JAK-3 is sufficient for efficacy in collagen-induced arthritis in mice. Arthritis Rheum 62: 2283–2293. 20506481 10.1002/art.27536

[44] O'Shea JJ, Plenge R (2012) JAK and STAT signaling molecules in immunoregulation and immune-mediated disease. Immunity 36: 542–550. 22520847 10.1016/j.immuni.2012.03.014 PMC3499974

[45] Maeshima K, Yamaoka K, Kubo S, Nakano K, Iwata S, et al (2012) The JAK inhibitor tofacitinib regulates synovitis through inhibition of interferon-gamma and interleukin-17 production by human CD4+ T cells. Arthritis Rheum 64: 1790–1798. 22147632 10.1002/art.34329

[46] LaBranche TP, Jesson MI, Radi ZA, Storer CE, Guzova JA, et al (2012) JAK inhibition with tofacitinib suppresses arthritic joint structural damage through decreased RANKL production. Arthritis Rheum 10.1002/art.34649 22899318

[47] Sakaguchi S, Benham H, Cope AP, Thomas R (2012) T-cell receptor signaling and the pathogenesis of autoimmune arthritis: insights from mouse and man. Immunol Cell Biol 90: 277–287. 22418389 10.1038/icb.2012.4

[48] Zhernakova A, Stahl EA, Trynka G, Raychaudhuri S, Festen EA, et al (2011) Meta-analysis of genome-wide association studies in celiac disease and rheumatoid arthritis identifies fourteen non-HLA shared loci. PLoS Genet 7: e1002004. 21383967 10.1371/journal.pgen.1002004 PMC3044685

[49] Abreu JR, Krausz S, Dontje W, Grabiec AM, de Launay D, et al (2010) Sustained T cell Rap1 signaling is protective in the collagen-induced arthritis model of rheumatoid arthritis. Arthritis Rheum 62: 3289–3299. 20662068 10.1002/art.27656

[50] Olasz K, Boldizsar F, Kis-Toth K, Tarjanyi O, Hegyi A, et al (2012) T cell receptor (TCR) signal strength controls arthritis severity in proteoglycan-specific TCR transgenic mice. Clin Exp Immunol 167: 346–355. 22236012 10.1111/j.1365-2249.2011.04506.x PMC3278702

[51] Malemud CJ (2011) Myeloid-related protein activity in rheumatoid arthritis. Int J Inflam 2011: 580295. 21876832 10.4061/2011/580295 PMC3157825

[52] Smith MD, Slavotinek J, Au V, Weedon H, Parker A, et al (2001) Successful treatment of rheumatoid arthritis is associated with a reduction in synovial membrane cytokines and cell adhesion molecule expression. Rheumatology (Oxford) 40: 965–977. 11561106 10.1093/rheumatology/40.9.965

[53] Klimiuk PA, Fiedorczyk M, Sierakowski S, Chwiecko J (2007) Soluble cell adhesion molecules (sICAM-1, sVCAM-1, and sE-selectin) in patients with early rheumatoid arthritis. Scand J Rheumatol 36: 345–350. 17963163 10.1080/03009740701406460

[54] Hunt KA, Mistry V, Bockett NA, Ahmad T, Ban M, et al (2013) Negligible impact of rare autoimmune-locus coding-region variants on missing heritability. Nature 10.1038/nature12170 PMC3736321 23698362

[55] Elbers CC, van Eijk KR, Franke L, Mulder F, van der Schouw YT, et al (2009) Using genome-wide pathway analysis to unravel the etiology of complex diseases. Genetic Epidemiology 33: 419–431. 19235186 10.1002/gepi.20395

[56] Jia P, Wang L, Meltzer HY, Zhao Z (2011) Pathway-based analysis of GWAS datasets: effective but caution required. Int J Neuropsychopharmacol 14: 567–572. 21208483 10.1017/S1461145710001446

[57] Maxwell JR, Gowers IR, Kuet KP, Barton A, Worthington J, et al (2012) Expression of the autoimmunity associated TNFAIP3 is increased in rheumatoid arthritis but does not differ according to genotype at 6q23. Rheumatology 51: 1514–1515. 22623711 10.1093/rheumatology/kes134

[58] Matmati M, Jacques P, Maelfait J, Verheugen E, Kool M, et al (2011) A20 (TNFAIP3) deficiency in myeloid cells triggers erosive polyarthritis resembling rheumatoid arthritis. Nature Genetics 43: 908–912. 21841782 10.1038/ng.874

[59] Daha NA, Kurreeman FA, Marques RB, Stoeken-Rijsbergen G, Verduijn W, et al (2009) Confirmation of STAT4, IL2/IL21, and CTLA4 polymorphisms in rheumatoid arthritis. Arthritis and Rheumatism 60: 1255–1260. 19404967 10.1002/art.24503

[60] Hollis-Moffatt JE, Chen-Xu M, Topless R, Dalbeth N, Gow PJ, et al (2010) Only one independent genetic association with rheumatoid arthritis within the KIAA1109-TENR-IL2-IL21 locus in Caucasian sample sets: confirmation of association of rs6822844 with rheumatoid arthritis at a genome-wide level of significance. Arthritis Res Ther 12: R116. 20553587 10.1186/ar3053 PMC2911910

[61] Knevel R, de Rooy DP, Zhernakova A, Grondal G, Krabben A, et al (2013) Association of Variants in IL2RA With Progression of Joint Destruction in Rheumatoid Arthritis. Arthritis and Rheumatism 65: 1684–1693. 23529819 10.1002/art.37938

[62] Bryl E, Vallejo AN, Matteson EL, Witkowski JM, Weyand CM, et al (2005) Modulation of CD28 expression with anti-tumor necrosis factor alpha therapy in rheumatoid arthritis. Arthritis and Rheumatism 52: 2996–3003. 16200579 10.1002/art.21353

[63] Kasperkovitz PV, Verbeet NL, Smeets TJ, van Rietschoten JG, Kraan MC, et al (2004) Activation of the STAT1 pathway in rheumatoid arthritis. Annals of the Rheumatic Diseases 63: 233–239. 14962955 10.1136/ard.2003.013276 PMC1754903

[64] Ishizaki M, Muromoto R, Akimoto T, Ohshiro Y, Takahashi M, et al (2011) Tyk2 deficiency protects joints against destruction in anti-type II collagen antibody-induced arthritis in mice. International Immunology 23: 575–582. 21765170 10.1093/intimm/dxr057

[65] Corr M, Boyle DL, Ronacher L, Flores N, Firestein GS (2009) Synergistic benefit in inflammatory arthritis by targeting I kappaB kinase epsilon and interferon beta. Annals of the Rheumatic Diseases 68: 257–263. 18653628 10.1136/ard.2008.095356 PMC2713581

