# Supplementary material for: Measles Contributes to Rheumatoid Arthritis: Evidence from Pathway and Network Analyses of Genome-Wide Association Studies
Source: PLoS One. 2013 Oct 18;8(10):e75951. doi: 10.1371/journal.pone.0075951 (PMC3799991; doi:10.1371/journal.pone.0075951)
Supplement: Table S1 — The detailed results from pathway analysis of RA GWAS. (DOC) [file pone.0075951.s001.doc]

Table S1.The detailed results from pathway analysis of RA GWAS

| Pathway Names | Pathway ID | Support | Reference | *P* | Genes Names |
| --- | --- | --- | --- | --- | --- |
| Kegg:05162 | Measles | 14 | 130 | 1.57E-08 | CDK6,STAT1,TNFAIP3,CDK4,TYK2,HSPA6,PRKCQ,TRAF6,  IL2RA,BBC3,IKBKE,CD209,CLEC4M,CD28 |
| Kegg:04660 | T cell receptor signaling pathway | 11 | 107 | 1.28E-06 | CDK4,PRKCQ,CD247,CTLA4,RAF1,TEC,ICOS,PTPRC,KRAS,LAT,  CD28 |
| Kegg:04060 | Cytokine-cytokine receptor interaction | 15 | 259 | 5.75E-06 | KIT,IL15,CNTFR,CD40,CCL19,IL6R,CXCR5,TNFRSF14,IL2RA,FLT3,  CCR5,IL11RA,CCL21,CCL27,CCR6 |
| Kegg:05223 | Non-small cell lung cancer | 7 | 53 | 4.57E-05 | CDK6,TGFA,ERBB2,CDK4,RASSF5,RAF1,KRAS |
| Kegg:05152 | Tuberculosis | 11 | 172 | 6.32E-05 | STAT1,RAB7A,CYP27B1,CASP8,FCGR2A,TRAF6,RAF1,FCGR3A,  CD209,CLEC4M,FCGR3B |
| Kegg:04630 | Jak-STAT signaling pathway | 10 | 153 | 1.25E-04 | STAT1,IL15,CNTFR,TYK2,STAT4,STAT6,IL6R,SPRED2,IL2RA,  IL11RA |
| Kegg:04672 | Intestinal immune network for IgA production | 6 | 44 | 1.25E-04 | IL15,CD40,ICOS,CD28,CCL27,PIGR |
| Kegg:04514 | Cell adhesion molecules (CAMs) | 9 | 125 | 1.26E-04 | PVRL1,CD40,CTLA4,ICAM1,ICOS,PTPRC,ICAM3,CD2,CD28 |
| Kegg:05212 | Pancreatic cancer | 7 | 70 | 1.36E-04 | CDK6,STAT1,TGFA,ERBB2,CDK4,RAF1,KRAS |
| Kegg:05200 | Pathways in cancer | 14 | 324 | 1.38E-04 | CDK6,STAT1,KIT,TGFA,FOXO1,ERBB2,CASP8,CDK4,RASSF5,  TRAF6,RAF1,FLT3,KRAS,TRAF1 |
| Kegg:04640 | Hematopoietic cell lineage | 7 | 83 | 3.42E-04 | KIT,IL6R,IL2RA,FLT3,CD19,IL11RA,CD2 |
| Kegg:04650 | Natural killer cell mediated cytotoxicity | 8 | 125 | 6.26E-04 | CD247,ULBP3,ICAM1,RAF1,FCGR3A,KRAS,LAT,FCGR3B |
| Kegg:05150 | Staphylococcus aureus infection | 5 | 46 | 1.23E-03 | C5,FCGR2A,ICAM1,FCGR3A,FCGR3B |
| Kegg:04062 | Chemokine signaling pathway | 9 | 186 | 1.62E-03 | STAT1,CCL19,CXCR5,RAF1,CCR5,KRAS,CCL21,CCL27,CCR6 |
| Kegg:05322 | Systemic lupus erythematosus | 6 | 88 | 2.84E-03 | CD40,C5,FCGR2A,FCGR3A,CD28,FCGR3B |
| Kegg:04380 | Osteoclast differentiation | 7 | 126 | 2.89E-03 | STAT1,TYK2,FCGR2A,TRAF6,TEC,FCGR3A,FCGR3B |
| Kegg:05145 | Toxoplasmosis | 7 | 123 | 2.99E-03 | STAT1,CD40,CASP8,TYK2,HSPA6,TRAF6,CCR5 |
| Kegg:05340 | Primary immunodeficiency | 4 | 33 | 3.02E-03 | CD40,ICOS,PTPRC,CD19 |
| Kegg:05214 | Glioma | 5 | 63 | 3.74E-03 | CDK6,TGFA,CDK4,RAF1,KRAS |
| Kegg:05140 | Leishmaniasis | 5 | 66 | 4.40E-03 | STAT1,FCGR2A,TRAF6,FCGR3A,FCGR3B |
| Kegg:04620 | Toll-like receptor signaling pathway | 6 | 101 | 4.51E-03 | STAT1,CD40,CASP8,IRF5,TRAF6,IKBKE |
| Kegg:04622 | RIG-I-like receptor signaling pathway | 5 | 70 | 5.23E-03 | CASP8,ATG5,TRAF6,IKBKE,AZI2 |
| Kegg:05219 | Bladder cancer | 4 | 42 | 5.67E-03 | ERBB2,CDK4,RAF1,KRAS |
